# Origin of Rashba Spin-Orbit Coupling in 2D and 3D Lead Iodide Perovskites

**DOI:** 10.1038/s41598-020-61768-8

**Published:** 2020-03-18

**Authors:** Minh T. Pham, Eric Amerling, Hoang M. Luong, Huy T. Pham, George K. Larsen, Luisa Whittaker-Brooks, Tho D. Nguyen

**Affiliations:** 1 Department of Physics & Astronomy, University of Georgia, Athens, GA 30602 USA; 2 Department of Chemistry, University of Utah, Salt Lake City, UT 84112 USA; 3 Department of Materials Science and Engineering, Phenikaa University, Ha Dong, Hanoi 10000 Vietnam; 4 National Security Directorate, Savannah River National Laboratory, Aiken, South Carolina 29808 USA

**Keywords:** Electronic devices, Spintronics

## Abstract

We studied spin dynamics of charge carriers in the superlattice-like Ruddlesden-Popper hybrid lead iodide perovskite semiconductors, 2D (BA)_2_(MA)Pb_2_I_7_ (with MA = CH_3_NH_3_, and BA = CH_3_(CH_2_)_3_NH_3_), and 3D MAPbI_3_ using the magnetic field effect (MFE) on conductivity and electroluminescence in their light emitting diodes (LEDs) at cryogenic temperatures. The semiconductors with distinct structural/bulk inversion symmetry breaking, when combined with colossal intrinsic spin–orbit coupling (SOC), theoretically give rise to giant Rashba-type SOC. We found that the magneto-conductance (MC) magnitude increases monotonically with the emission intensity and saturates at ≈0.05% and 0.11% for the MAPbI_3_ and (BA)_2_(MA)Pb_2_I_7_, respectively. The magneto-electroluminescence (MEL) response with similar line shapes as the MC response has a significantly larger magnitude, and essentially stays constant at ≈0.22% and ≈0.20% for MAPbI_3_ and (BA)_2_(MA)Pb_2_I_7_, respectively. The sign and magnitude of the MC and MEL responses can be quantitatively explained in the framework of the Δg-based excitonic model using rate equations. Remarkably, the width of the MEL response in those materials linearly increases with increasing the applied electric field, where the Rashba coefficient in (BA)_2_(MA)Pb_2_I_7_ is estimated to be about 7 times larger than that in MAPbI_3_. Our studies might have significant impact on future development of electrically-controlled spin logic devices via Rashba-like effects.

## Introduction

**Figure 1:**
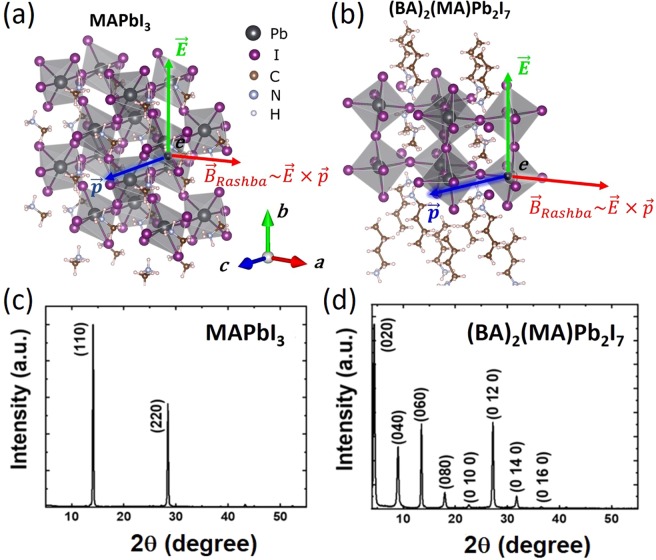
Crystal structures for 2D/3D superlattice-like Ruddlesden-Popper hybrid lead iodide perovskite semiconductors, (BA)_2_ (MA)_n−1_Pb_n_I_3n+1_ with (**a**) n = ∞ (3D MAPbI3), and (**b**) n = 2 (2D (BA)2(MA)Pb2I7). In their LEDs, the applied electric field is in the vertical direction causing ***B***_***Rashba***_ field. X-ray diffraction spectra for (**c**) 3D MAPbI_3_ and (**d**) 2D (BA)_2_(MA)Pb_2_I_7_ thin films.

Organic-inorganic hybrid perovskites (OIHPs) are rapidly emerging as functional materials for novel optoelectronic and quantum electronic devices^1,2^. In these materials, strong intrinsic spin-orbit coupling (SOC) stemming from the heavy metal atoms in the inorganic framework (i.e., Pb or Sn) plays a decisive role in significantly lowering the optical band gap down to near the infrared region and preserving optical absorption from perturbations by local distortions of the lattice. These effects have been harnessed to produce high performance solar cells^3^. In fact, 3D methylammonium lead iodide (MAPbI_3_) OIHP is an excellent material for optoelectronics due to its superb physical properties such as large dielectric constant of above 10^4,5^, long carrier diffusion lengths on the order of 100 μm^6^, high electron/hole mobility of ≈100 cm^2^(Vs)^−1^ ^7^, large absorption coefficient as high as 10^4^  cm^−1^^1^, and small exciton binding energy of a few meV at room temperature^5,8^. Recently, the power conversion efficiency for a single-junction OIHP solar cell having an analog of MAPbI_3_ as the absorber layer exceeded 25%^9^. The high power conversion efficiency observed for these materials may be related to the degree of SOC strength present in their crystal structure. It has been hypothesized that the strong SOC in the absence of inversion symmetry in OIHPs gives rise to Rashba-type effects, where spin-dependent properties can be manipulated by electric fields^10–13^. As a consequence, the spin degeneracy in k-space is lifted and the valence band maxima and/or conduction band minima of up- and down-spin states are shifted away from the symmetry points in the Brillouin zone triggering an indirect bandgap transition in the materials^3,12–15^. Indirect bandgap transitions due to Rashba effects have been experimentally characterized by time-resolved photoconductivity measurements^16^ and polarization- and angle-resolved photoemission spectroscopy (ARPES)^17^, and have been further supported by recent observations of a circular photogalvanic effect^18^. Opto-electronically, the indirect energy-band structure in a material demonstrating Rashba effects leads to a forbidden indirect transition that suppresses electron-hole pair recombination rates, which is beneficial for high solar cell performance. Large Rashba coefficients on the order of several eV·Å have been measured by ARPES in 3D OIHPs^17^ and by electroabsorption spectroscopy in 2D OIHPs^19^. These results are very promising for spin-logic device applications^14^ such as spin field-effect transistors, in which a spin current is manipulated by an applied electric field^20–22^. The large Rashba coefficient observed in 3D MAPbI_3_ OIHP^15,23–25^ in the range from 1 to 4 eV·Å is attributed to the bulk inversion symmetry breaking that originates from the octahedral tilting of the inorganic lead-halide cage and the dynamic rotation of the organic cation on the timescale of a few picoseconds^26^ (see Fig. 1a, the chemical structure was taken from Ref. ^27^). A similar large Rashba coefficient of ≈1.6 eV·Å has been recently observed in 2D OIHPs based on phenethylammonium lead iodide -(C_6_H_5_C_2_H_4_NH_3_)_2_PbI_4_ and has been attributed to the inversion asymmetry of the crystal structure^19^ (see Fig. 1b, the chemical structure was taken from Ref. ^28^). Interestingly, the inversion asymmetry of these 2D and 3D OIHPs can be estimated by second-harmonic generation measurement, where 2D OIHPs show much stronger second-harmonic generation values than their 3D counterparts^28^. These results are intriguing, but such an experiment does not directly probe the Rashba interaction energies in those materials. Yu^29^ calculated the Rashba coefficients in the 2D and 3D OIHOs using the structural inversion symmetry breaking of each materials taken from the second-harmonic generation experimental results. He showed that the Rashba coefficient in the analogous 2D OIHP is about 8 times larger than that in 3D MAPbI_3_ OIHP, contrary to the above mentioned reports that showed their Rashba coefficients to be similar^29^.

To the best of our knowledge, a direct comparison of the Rashba effect under the influence of an applied electric field, and the spin interaction in general of 2D and 3D OIHPs has not been experimentally demonstrated. One way to perform such a comparison is to study the magnetic field effect (MFE) in light emitting diodes (LED) constructed of those OIHP materials. We note that although the spin dynamics in organic semiconductors have been extensively studied using MFE in organic LEDs^30–35^, such MFE studies have been rarely used to investigate the spin response in 3D and 2D OIHPs. In contrast to the second harmonic generation study of Rashba SOC, the applied magnetic field in the MFE method directly manipulates the spin precession of charge carriers and alters the spin mixing rate between loosely bound singlet and triplet excitons due to their high dielectric screening^4,5^. This is fundamentally different from spin mixing mechanism in organic semiconductors where the magnetic field only acts on the loosely bound electron-hole pairs, not excitons with much larger exciton binding energy. Consequently, both electroluminescence (EL) intensity and conductivity in OIHP based LEDs are strongly modulated by the applied magnetic field. The half width at half maximum (HWMH) of the magneto-electroluminescence (MEL) and magneto-conductance (MC) are considered to be proportional to spin-interaction energy in the emissive OIHP layer of the LED. It is worth noting that spin interaction in OIHP materials is also very different from that in conventional organic semiconductors, where the hyperfine interaction is usually dominant and Rashba type SOC is typically negligible. So far, only a few investigations of MFE in photoconductivity and electroluminescence in OIHPs exist in the literature, and none which investigate the effect of Rashba SOC on the conductivity of OIHPs^36,37^.

In this work, we probed the spin dynamics of charge carriers in solution-processed superlattice-like Ruddlesden-Popper OIHP semiconductors ((BA)_2_(MA)_n−1_Pb_n_I_3n+1_ where n = 1, 2, 3, 4, ∞)^28^, butyl- methyl-ammonium lead iodide (BA)_2_(MA)Pb_2_I_7_ (n = 2) and MAPbI_3_ (n = ∞) nanocrystals using the MFE on conductivity and EL in their LEDs at cryogenic temperatures. The OIHPs studied here are very different in the structural inversion asymmetry (Fig. 1a,b). An applied electric field along the structural asymmetric direction of the (BA)_2_(MA)Pb_2_I_7_ OIHP strongly modifies the local electric field, ***E*** inside the material. On the other hand, since MAPbI_3_ OIHP with the bulk inverstion assymmetry shows strong ferroelectric characteristics^38^, ***E*** in the material is also expected to be changed upon the applied electric field. As a result, the effective magnetic field (Rashba field), ***B***_***Rashba***_ on charge carriers moving with momentum, ***p*** in the direction perpendicular to ***E*** can be described as ${{\boldsymbol{B}}}_{{\boldsymbol{Rashba}}}\propto {\boldsymbol{E}}\times {\boldsymbol{p}}$ (see Fig. 1a,b**)** and is strongly modulated by an applied electric field. Such electric field dependent ***B***_***Rashba***_ may have a sizable effect on the spin dynamics/configuration of electrons and holes, and therefore, influences the conductivity and electroluminescence in the device. In order to quantify these effects, the MFE response of OIHP based LEDs was used to estimate the magnitude of the SOC strength, effective local magnetic field, and Rashba SOC/field as a function of applied electric fields.

## Results and discussions

Figure 1a depicts the crystal structure for 3D MAPbI_3_. Here, the PbI_6_ octahedral bond together form an isotropic tetrahedral cage surrounded by the organic cations (MA^+^). In contrast, the quasi-2D (BA)_2_(MA)Pb_2_I_7_ quantum well structure (Fig. 1b) can be obtained by slicing the 3D MAPbI_3_ tetragonal structure along the 〈110〉 planes and replacing the MA^+^ cation with a bulky BA^+^ organic cation. As depicted in Fig. 1b, there are two layers of PbI_6_ with bulky BA^+^ cations on either side of the layers that are held together by weak van der Waals forces. Thin films based on synthesized 3D MAPbI_3_ and 2D (BA)_2_(MA)Pb_2_I_7_ materials were fabricated via spin-coating methods yielding thicknesses of 50 nm and 130 nm, respectively, which were found to be the optimal dimensions for high quality films and devices (See Methods Section).

The structures have been confirmed by X-ray diffraction (XRD) spectral studies at room temperature as described in Fig. 1c,d^28^. The crystallographic plane assigned for each diffraction peak is in agreement with previous reports^28^. In the case of MAPbI_3_ thin films, the high-quality material only displays peaks from the {hk0} family of reflections, providing evidence of a well oriented thin film (Fig. 1c)^39^. In the case of (BA)_2_(MA)Pb_2_I_7_ thin films, the XRD profile only shows diffraction peaks associated with the {0k0} family of crystallographic planes (Fig. 1d). This suggests the formation of highly oriented inorganic sheets parallel to the substrate. Moreover, the diffraction peaks obtained for (BA)_2_(MA)Pb_2_I_7_ films are much stronger and narrower than those observed for MAPBI_3_ films. This indicates that the crystal domain size of the 2D OIHP thin films is larger than of 3D OIHP thin films. Using the Scherrer’s equation, we calculated the crystal domain sizes of the 3D MAPbI_3_ and 2D (BA)_2_(MA)Pb_2_I_7_ crystal, and these are 38.1 and 44.9 nm, respectively (see Table S1 Supporting Information). We note that the MAPbI_3_ shows the tetragonal phase at room temperature while the MFE was studied in its orthorhombic phase at 10 K^39^.

**Figure 2:**
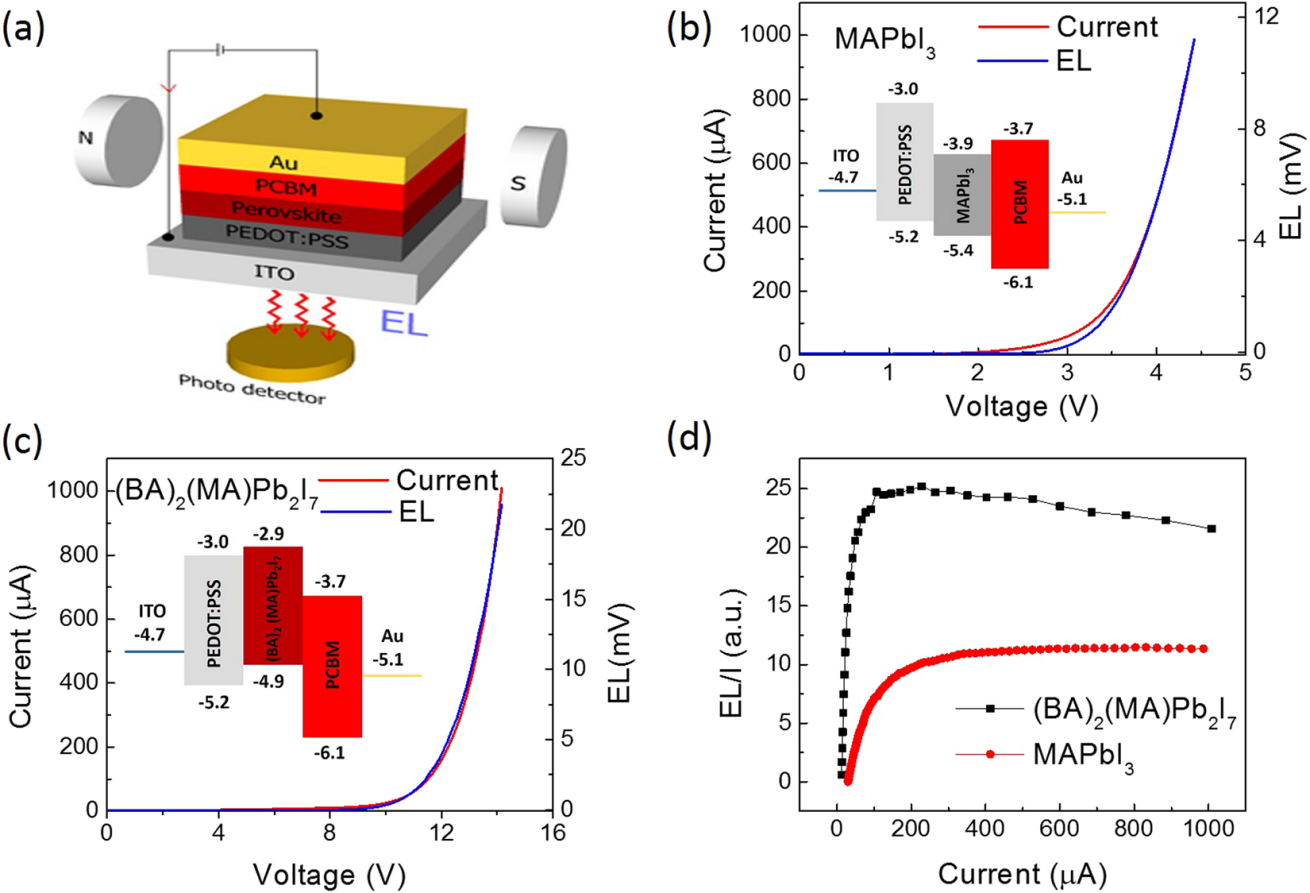
(**a**) Light emitting diodes (LED) based on 3D and 2D OIHPs. The current-voltage (I-V) and electroluminescence-voltage (EL-V) characteristics for 3D and 2D OIHP based LEDs are shown in (**b**,**c**) at 10 K. The insets in (**b**,**c**) show the energy diagram of the devices. (**d**) EL quantum efficiency of the LEDs.

Figure 2b,c respectively show the current-voltage (I-V) and electroluminescence-voltage (EL-V) for MAPbI_3_ and (BA)_2_(MA)Pb_2_I_7_ based LEDs as described in Fig. 2a (see the Method section for device fabrication and measurement). The spin dynamics of phase-pure OIHPs were investigated at 10 K because the luminescence response is much stronger at low temperatures than at room temperature. The turn-on voltage for the EL (at ≈2.3 V and ≈10 μA) in 3D MAPbI_3_ LED is lower than that observed in 2D (BA)_2_(MA)Pb_2_I_7_ LED (at ≈8.6 V and ≈12 μA) mainly because the (BA)_2_(MA)Pb_2_I_7_ LED has a thicker emissive layer (≈130 nm) than that in MAPbI_3_ LED (≈50 nm)^40^. In addition, the larger band-gap in (BA)_2_(MA)Pb_2_I_7_ IOHP (≈2.0 eV in comparison to ≈1.5 eV in the MAPbI_3_ IOHP) also results in the higher EL turn-on voltage. Finally, the low out-of-plane conductivity in the (BA)_2_(MA)Pb_2_I_7_ LED may also play a detrimental role in charge transport across the highly oriented organic sheets due to the low hoping/tunneling charge mobility. We note that the devices have Ohmic contacts with hole-only transport at the voltage below the EL-turn on voltage (see Fig. S1 Supporting Information). However, the electron-hole recombination is quite strong at higher applied voltages.

The EL quantum efficiency (ELQE), which is proportional to the ratio of the EL intensity over the current density, is plotted in Fig. 2d. The ELQE in both devices monotonically increases from zero and saturates at a current density of ≈200 μA/mm^2^. However, the ELQE in 2D (BA)_2_(MA)Pb_2_I_7_ decreases at higher current densities. This slight ELQE reduction may be associated with exciton loss due to strong exciton-exciton annihilation when the dense excitons at high current densities are confined in a 2D well. Next, we will show MFE studies on the devices’ conductivity and EL intensity at different current densities in the Ohmic and exciton recombination regimes.

**Figure 3:**
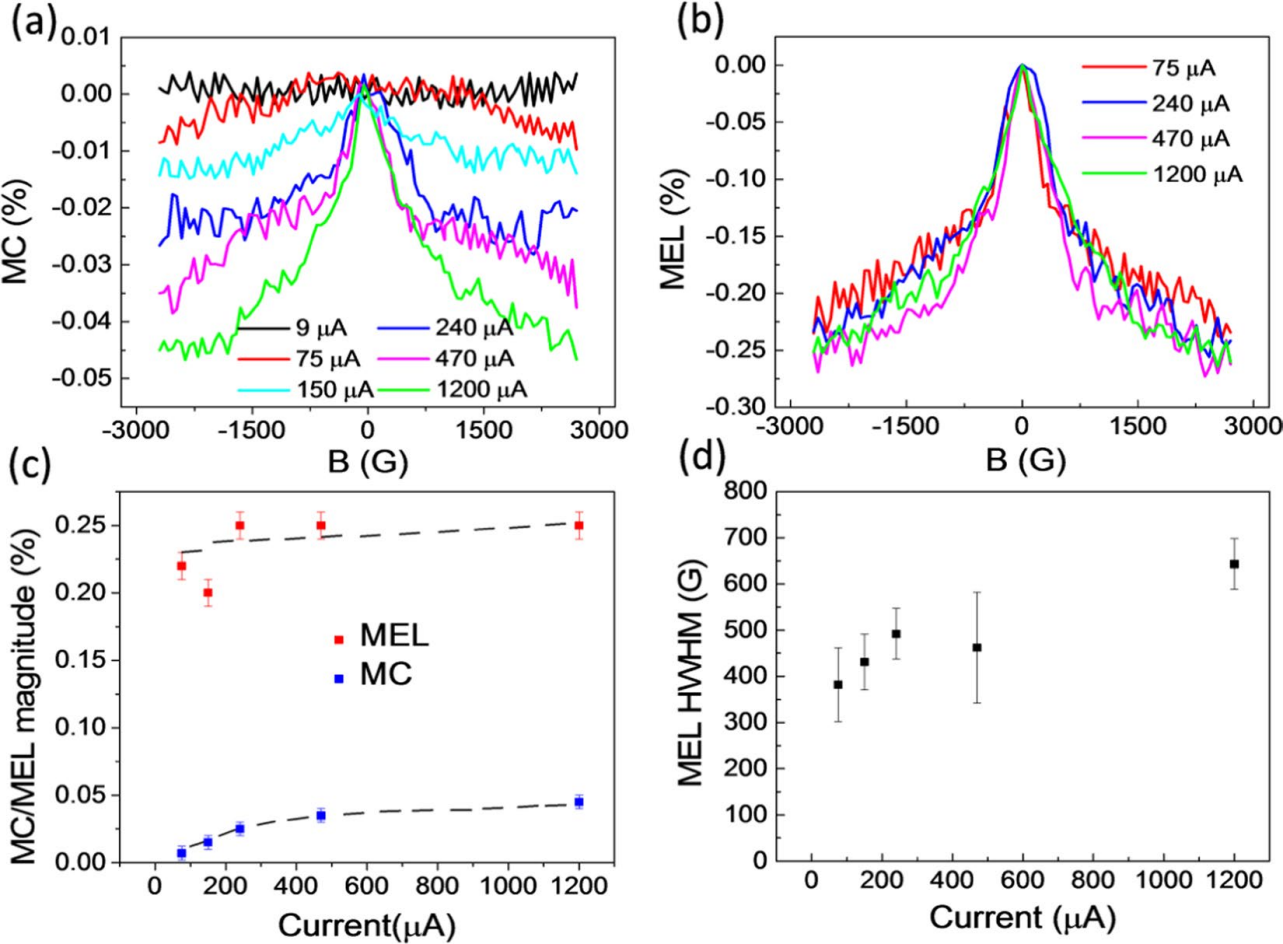
(**a**) Magneto-conductance (MC), and (**b**) magneto-electroluminescence (MEL) of a typical 3D MAPbI_3_ based LED at several device currents. (**c**) Extracted MC and MEL magnitudes versus device current. The lines are guides for the eyes. (**d**) The haft width at half maximum (HWHM) of MEL response extracted directly from (**b**) versus device currents.

Figure 3a shows the typical MC responses up to 3 kG at six different current densities for a 3D MAPbI_3_ LED, where the MC magnitude is found to increase with increasing current density and saturate at ≈200 μA. At a current of 75 μA, the MC is about (0.01 ± 0.005) % while at a current of 1200 μA, the MC reaches (0.05 ± 0.005) % at an applied field of 3 kG. We do not observe any MC response within 0.005% noise when the current density is below the turn-on current density for the EL (see MC at ≈9 μA/mm^2^ in Fig. 3a, and at ≈2 μA/mm^2^ in Fig. S2a). It is also important to note that the MC current noise level may distort the shape of the MC at low current densities. For example, the MC at 75 μA is less than 0.01%, which is only about 2 times larger than the noise level. Figure 3b shows the corresponding MEL responses at several current densities. In contrast to the MC response, the MEL response remains the same magnitude of ≈(0.22 ± 0.03) % at all current densities. MEL response at 150 μA can be seen in Fig. S2b. The magnitude of MC and MEL versus the device current extracted directly from Fig. 3a,b are plotted in Fig. 3c where the MC magnitude monotonically increases from zero and saturates at ≈200 μA, in agreement with studies of the magneto-photocurrent trend reported in Zhang *et al*.^36^ Interestingly, the MC magnitude trend is the same as the ELQE response as shown in Fig. 2d. In general, the MEL and MC responses look similar with an exception observed at low current density of 75 μA/mm^2^. This suggests that the MEL and MC share the same physical interpretation. Since the MFE is not observed when the EL is absent and the MEL magnitude is much larger than the MC magnitude, the magnetic field must primarily act on the EL, and MC is just a secondary effect, e.g. from electron-hole pair dissociation. This indicates that the MC response must have an excitonic origin where the intersystem crossing between singlet and triplet electron-hole pairs^36^ or triplet-charge interaction (or trion)^41^ may take place. We note that both mechanisms require the existence of trapped charges, and long exciton lifetimes to have sizable magnetic field effect^36^. Nevertheless, the triplet-charge interaction requires a long-lived triplet exciton in the ms range, typically found in organic semiconductors. This long-lived triplet exciton is unlikely to occur in OIHPs due to the exceptionally strong SOC. In fact, to the best of our knowledge, the trion formation has not been reported in OIHP thin films. Zhang *et al*. showed that electron-hole lifetimes of OIHP films is in the ps range and increases with trapped charges^36^. The authors found that the magneto-photocurrent is associated with the intersystem crossing rate between singlet-triplet excitons, which is not affected by the hyperfine interaction, but the so-called Δg mechanism. Remarkably, the half width at half maximum (HWHM) of the MEL response or effective local magnetic field in Fig. 3d slightly increases with increasing the current density (see Fig. S3 for the method to extract the width). This implies that the applied electric field might influence the spin mixing processes among the spin sublevels or the intersystem crossing rate between singlet and triplet electron-hole pairs via the Rashba effect^30,42,43^.

**Figure 4:**
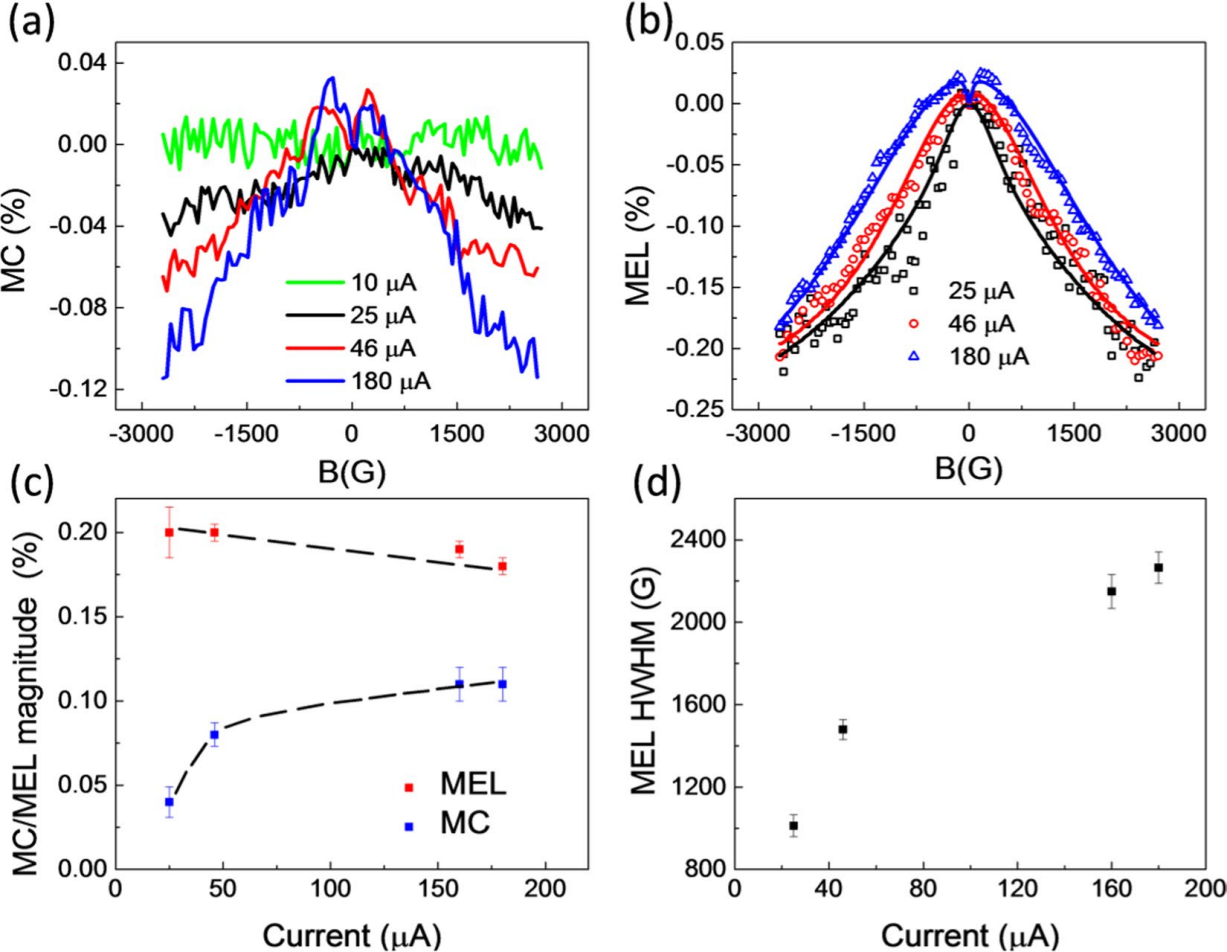
(**a**) Magneto-conductance (MC), and (**b**) magneto-electroluminescence (MEL) of a typical 2D (BA)_2_(MA)Pb_2_I_7_ based LED at 10, 25, 46, and 180 μA device currents. The lines show best fits with a double-Lorentzian function. (**c**) MC magnitude and MEL magnitude versus device currents. The lines are guides for the eyes. (**d**) The half at half maximum (HWHM) of the MEL response obtained from the MEL fits versus device currents.

Figure 4a,b show the MC and MEL responses for a 2D (BA)_2_(MA)Pb_2_I_7_ LED. Similar to the response for MAPbI_3_, the MC magnitude increases monotonically and saturates at ≈(0.11 ± 0.02)% while the MEL magnitude is larger and remains essentially the same at ≈(0.20 ± 0.02)% with increasing current density (see Fig. 4c). The MC and MEL responses at 160 μA are shown in Fig. S4b,c. The MC response is not detectable within 0.01% noise when the current density is smaller than 10 μA/mm^2^ (also see Fig. S4a). Both MC and MEL contain two components at small field and high field, which are usually observed in organic semiconductors with strong intrinsic SOC^43,44^. Because the MC magnitude versus the LED current follows the same trend as the ELQE trend shown in Fig. 2d, we conclude that MC in 2D (BA)_2_(MA)Pb_2_I_7_ LED device must have the same excitonic origin as the MC in the 3D MAPbI_3_ LED device. Since the MEL response of 2D (BA)_2_(MA)Pb_2_I_7_ is noisy and very broad, we are not able to saturate the response at an applied field of 3 kG. Instead, we fitted the data with a double-Lorentzian function (MC_1_*B^2^/(B^2^ + B_1_^2^) + MC_2_*B^2^/(B^2^ + B_2_^2^), where MC_1_, MC_2_, B_1_, B_2_ are fitting parameters. All fitting parameters are presented in Table S2, Supporting Information. The lines in Fig. 4b shows the fit result up to 3 kG while Fig. S5 shows the fit lines up to 20 kG. The HWHM of the MEL response extracted from Fig. S3 versus the LED current is plotted in Fig. 4d. The width nonlinearly increases with increasing current density. Clearly, the spin dynamics in 2D and 3D materials follow the same mechanism.

It has been extensively studied in organic semiconductors that strong SOC suppresses the MFE magnitude while broadening the HWHM^43,44^. The MFE studies in 2D and 3D OIHP based LEDs agree very well with those in organic semiconductors. In contrast, the MFE in OIHPs is insensitive to hyperfine interaction due to ultra-short exciton lifetime^36^. In addition to the intrinsic SOC, OIHPs may exhibit strong Rashba type SOC with exceptionally large Rashba coefficients due to the bulk and structural symmetry breaking as discussed in the introduction. Such properties allow the control the Rashba SOC strength, and hence spin dynamics, by an applied electric field^21,22,45^. Figs. 3d and 4d clearly show that the spin response becomes stronger at larger current density or larger applied voltage.

**Figure 5:**
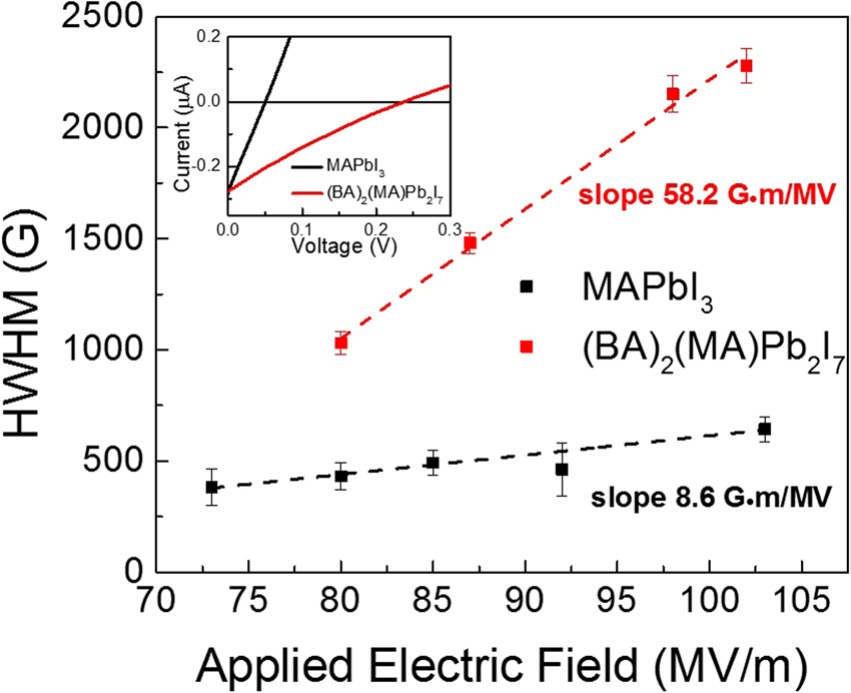
The HWHM of MEL response for 2D and 3D OIHP based LEDs versus applied electric field. The dashed lines show linear fits. The inset shows the photocurrent response of the devices.

To investigate the effect of Rashba SOC on the MEL response, we replotted the results presented in Figs. 3d and 4d as a function of applied electric field (see Fig. 1a,b for the applied field direction) rather than the current density and they are depicted in Fig. 5. It is reasonable to assume that the electric field drops are negligible across the PEDOT:PSS hole transport layer and PCBM electron transport thin layers^46^. Therefore the applied electric field, E, can be estimated by E = (V-V_OC_)/d, where V is the applied voltage, V_OC_ is the open circuit voltage which can be extracted from the photocurrent (see Fig. 5, inset) in which the I-V characteristics were taken under 450 nm laser illumination at 100 mW/cm^2^, and d is the thickness of the OIHP thin film. Interestingly, for both 3D and 2D OIHPs, the MEL HWHM increases linearly with the applied electric field. We fit the data with a linear function whose fitting parameters are shown in Fig. S6, Supporting Information. We found that the effective SOC energy (***B***_***Rashba***_) in the (BA)_2_(MA)Pb_2_I_7_ OIHP increases at a large rate of 58.2 G·m /MV in comparison with 8.6 G·m /MV for the MAPbI_3_ OIHP (Fig. 5). The applied electric field along the 2D wells in the (BA)_2_(MA)Pb_2_I_7_ OIHP clearly causes significant manipulation of local electric field, **E** and hence ***B***_***Rashba***_. Our results are consistent with the experimental results reported by Wang *et al*. who observed the linear dependence of Rashba SOC energy on the applied electric field in GaAs quantum wells^22^. The negative intercepts of the linear fits in Fig. S6 might tell us about the Dresselhaus and intrinsic SOC energy as analyzed by Wang *et al*. In the first approximation, this suggests that the Rashba coefficient in (BA)_2_(MA)Pb_2_I_7_ OIHP is about 7 times larger than the coefficient in MAPbI_3_ OIHP. These results are in agreement with recent calculation of Rashba SOC coefficients on those materials using the second harmonic generation experimental data, where the ratio of the Rashba coefficient in the (BA)_2_(MA)Pb_2_I_7_ OIHP over the MAPbI_3_ OIHP is about 8 times^29^. Clearly, the (BA)_2_(MA)Pb_2_I_7_ OIHP is more suitable for the electrically controlled spin logic device. Interestingly, the effect of electric field on the MC line shape in (BA)_2_(MA)Pb_2_I_7_ OIHP is essentially the same as the effect reported by Takase *et al*. who studied the highly gate-tunable Rashba spin-orbit interaction in InAs nanowire metal-oxide-semiconductor field-effect transistor^21^.

**Figure 6:**
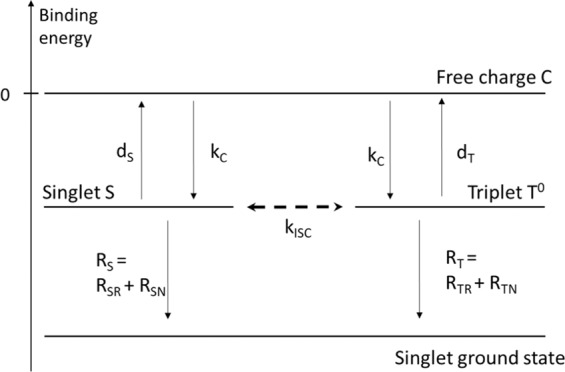
The schematic energy level diagram illustrates the simplest possible excitonic model, which includes three different species: free charges with population C, singlet electron-hole pairs with population S, and triplet electron-hole pairs with population T^0^. Various transition rates are indicated.

There are several mechanisms for the spin mixing event from the spin sublevels causing intersystem crossing between singlet and triplet electron-hole pairs. These include hyperfine interaction, SOC, exchange, and Δg mechanism. Since the spin relaxation time in OIHPs is very fast due to strong SOC, much slower spin precession caused by the hyperfine field cannot cause the spin mixing between the singlet and triplet electron-hole pairs^47^. We note that the magnetic field from the SOC including the Rashba-type SOC is significantly larger than the hyperfine field of ≈50 G^33,48^. The large SOC field might result in a fast spin precession time that is comparable to the exciton lifetime causing the MEL and MC. For the Δg mechanism, the large spin mixing rate, $\Delta {\omega }_{p}\propto B\Delta g$, is possible at large applied magnetic field, B, and different g factors between the electron and hole in the spin pair. A $\Delta g$ value of ≈1.0 in the OIHPs was found to be significantly larger than that in organic semiconductors (≈10^−3^)^36,49^. Therefore, the $\Delta g$ spin mixing is more likely a dominant mechanism in OIHPs. Because the $\Delta g$ in 2D (BA)_2_(MA)Pb_2_I_7_ OIHPs $(\Delta g\approx 1.2)$ is found to be smaller than that in 3D MAPbI_3_ OIHPs $(\Delta g\approx 1.7)$^49^, the spin mixing in (BA)_2_(MA)Pb_2_I_7_ OIHPs would be slower than that in MAPbI_3_ OIHPs. Therefore, we would expect to have a broader MFE response in (BA)_2_(MA)Pb_2_I_7_ based LEDs. The Rahsba energy increases with increasing applied electric field in our experimental results in Fig. 5 implying that the $\Delta g$ value must be smaller under a larger applied electric field or larger ***B***_***Rashba***_. We note that such a model has been used to explain the MFE in photocurrent, which is a different concept^36^. Next, we will examine MFE using the excitonic electron-hole pair model that has been successfully applied to explain the MFE in organic LEDs. It is therefore natural to ask whether this model can explain the sign and the magnitude of MC and MEL responses in OIHPs. The electron-hole pair dynamics can be described as following: When electrons and holes are injected from the cathode and anode into the organic layer, they form negative and positive charges, respectively. When the separation between an electron and a hole is less than the Coulomb capture radius, the carriers are organized in loosely bound electron-hole pairs with the rate *k*_*C*_, statistically 25% spin singlet pair *S with zero spin*, 75% spin triplet pairs *T* ^0^,*T*^1^, and *T*^−*1*^ where 0, 1 and -1 is the spin triplet multiplicity. Since the electrons and holes in OIHPs are highly delocalized due to the dielectric screening, electrons and holes in the excitonic form are loosely bound with an excitonic binding energy of a few meV^4,5^. In the first approximation, the exchange interaction energy between singlet and triplet electron-hole pair is negligible; therefore the singlet and triplet excitons essentially stay in the same energy level. Finally, the singlet and triplet electron-hole pairs can recombine with the rate *R*_*S*_ and *R*_*T*_ or dissociate to form free charges (C) with the rate *d*_*S*_ and *d*_*T*_, respectively. The rate *R*_*S*_ (*R*_*T*_) might include radiative recombination with the rate *R*_*SR*_ (*R*_*TR*_) or non-radiative recombination with the rate *R*_*SN*_ (*R*_*TN*_). We can now formulate appropriate rate equations. The relevant energy levels and transition rates between them are shown in Fig. 6.

The basic idea of the exitonic model is that the multiplicity of the singlet and triplet electron-hole pairs/excitons changes with time due to spin dynamics induced by either the Δg mechanism or SOC. While the SOC can mix all triplet excitons with singlet excitons, the non-zero difference in the g-factor of electrons and holes, *Δg*, leads to the spin mixing rate, *k*_*ISC*_ between the isoenergetic *S* and *T* ^0^. Since Δg is significantly larger in OIHPs, the Δg effect is more likely to play a crucial role in the observed Rashba effect. Therefore, the schematic energy diagram with relavent energy levels descibed in Fig. 6 is examined. Several rate equations based on the Δg mechanism can be written from the diagram in Fig. 6 as following:

1 $$-{k}_{C}C+{d}_{S}S+{d}_{T}{T}^{0}={G}_{C}$$

2 $${k}_{C}C-({d}_{S}+{R}_{S}+{k}_{ISC})S+{k}_{ISC}{T}^{0}=0$$

3 $${k}_{C}C+{k}_{ISC}S-({d}_{T}+{R}_{T}+{k}_{ISC}){T}^{0}=0$$

where *G*_*C*_ is the generation rate for *C*, which is equal to the rate of carrier injection minus the rate of emission of carriers at the electrodes. At zero applied magnetic field, it is obvious that *k*_ISC_ = 0. At large applied magnetic fields, we suppose that the k_ISC_ is much larger than other transition rates. We suppose *d*_*S*_ *= d*_*T*_, the LED current ≈ *C*, and $EL={R}_{SR}S+{R}_{TR}T\,\approx \,{R}_{SR}S$ for most OIHPs^50^. We obtain the following solutions from the rate Eqs. (1–3):

4 $$MEL=\frac{(1-{R}_{S}/{R}_{T})}{\left(1+\frac{{R}_{S}}{{R}_{T}}\right)({d}_{S}/{R}_{T}+1)}$$

5 $$MC=\eta \,\frac{-{(1-{R}_{S}/{R}_{T})}^{2}}{2(1+{R}_{S}/{R}_{T})(1+{R}_{S}/ds)({d}_{S}/{R}_{T}+1)},$$

where *η* is the fraction of the injected carriers that form electron-hole pairs. In general, MEL and MC is non-zero if the recombination rate of singlet and triplet are different. Now, the MC and MEL responses in Figs. 3 and 4 can be examined using Eqs. (4) and (5). The magnitude of MC is significantly smaller than MEL because of the following reasons. First, MC is a second order effect, while MEL appears in first order in (1-R_S_/ R_T_). If R_S_ ≈ R_T_, MEL magnitude is generally larger than MC magnitude. Next, because the applied magnetic field does not act on the non-emissive excitons, *T*^1^ and *T*^−*1*^_,_ free charge carriers dissociated from these states do not contribute to increasing, but decreasing the MC magnitude. In fact, the MC effect is approximately about 50% smaller than that in Eq. (5), while MEL in the first order approximation is unaffected by the dissociation/recombination of these dark exciton states. Finally, *η* in our device structures is small due to the imbalance of positive/negative injected carriers and the small Coulomb binding energy. The small value of *η* may lead to a significantly small MC. Considering the sign of the MC and MEL effect, the model predicts that MC is always negative while MEL is more likely to be negative since ${R}_{S}\,{\rm{is}}\,{\rm{usually}}\,{\rm{large}}\,{\rm{then}}\,{R}_{T}$ for most OIHPs^50^, in agreement with the experimental results.

In conclusion, we studied the Rashba-type SOC in 2D (BA)_2_(MA)Pb_2_I_7_ and 3D MAPbI_3_ OIHPs using the MFE on the conductivity and the EL. We found that the MC magnitude in LEDs constructed with these OIHPs increases monotonically and saturates at a large current density. The MC follows the same trend as the ELQE of the device, while the MEL response remains almost unchanged and has similar shapes as the MC response, but with a significantly larger magnitude. This implies that the underlying mechanism of the MFE in the materials is excitonic. Notably, we found that the width of the MEL, or ***B***_***Rashba***_, in these materials linearly increases with increasing the applied electric field. The Rashba coefficient in (BA)_2_(MA)Pb_2_I_7_ OIHPs is estimated to be about 7 times larger than that in MAPbI_3_ OIHPs. An excitonic model was developed to evaluate the MFE mechanism. We found that both sign and magnitude of MEL and MC responses can be explained through this model. An important prediction of our experimental results is that the $\Delta g$ must be smaller under the effect of an applied electric field.

## Methods

All the chemical reactions were performed in a controlled atmosphere. Lead (II) oxide (PbO, ≥99.0% pure), n-butylamine (CH_3_(CH_2_)_3_NH_2_; 99.5% pure), and stabilized hydriodic acid (HI; 57 wt. % in H_2_O, contains ≤1.5% H_3_PO_4_ acid as a stabilizer) were purchased from Sigma Aldrich and used without further purification. Methylammonium chloride (MACl) was purchased from Dyesol (now Greatcell Solar) and used without further purification.

For the MAPbI_3_ (n = ∞), 4.8 mmol (1.1 g) of PbO was dissolved in 10 mL of stabilized HI. The solution was then heated at 60 °C and stirred until completely dissolved and brilliantly yellow. Then, 7.2 mmol (500 mg) of MACl was added to the solution which turned black instantly. The solution was left to stir for 30 min. Subsequently, a black precipitate was formed. Prior to any characterization, this precipitate was washed with cold ether and dried in a vacuum oven at 60 °C for 2 h.

For the (BA)_2_MAPb_2_I_7_ crystals (n = 2), 4.8 mmol (1.1 g) of PbO was dissolved in 10 mL of stabilized HI, heated mildly (60 °C), and stirred until completely dissolved and brilliantly yellow. Then, 2.4 mmol (162 mg) of MACl was added to the solution and instantly turned black. After a few minutes of heating and stirring, the MACl was completely dissolved and the solution returned to a bright yellow state. Next, 4.8 mmol (480 μL) of n-butylamine was added to the solution dropwise under vigorous stirring. The solution was left to stir for 10 min. A 3:1 addition of NaI:PbI_2_ (2.2 g) was added to prevent decomposition and formation of defective perovskite phases. Finally, 6 mL of glacial acetic acid was added to quench the reaction and form the desired product. This reaction yields red crystals which were filtered and washed with cold ether three times before being placed in a vacuum oven at 60 °C for 2 h.

The 2D and 3D OIHP powders were dissolved in a dimethylformamide (DMF) solution to yield a 0.25 M concentration.

The LED architecture is depicted in Fig. 2a. The fabrication of the organic sandwich devices started with patterning 40 nm of indium-tin-oxide (ITO) on a glass substrate using photolithography and chemical etching. Then, the ITO-coated glass substrate was thoroughly cleaned with soap, distilled water, acetone, and isopropanol in an ultrasonic bath. The conducting polymer poly(3,4-ethylenedioxythiophene)-poly (styrenesulfonate) (PEDOT) from Ossila was spin coated at 4000 rpm for 60 s on top of the ITO-coated glass substrate yielding a thin PEDOT layer of ≈20 nm thick to provide an efficient hole injecting electrode. All other fabrication steps were carried out in a nitrogen filled glovebox. Inside the glovebox, the perovskite solutions and glass substrates were preheated to 80 °C for 30 min. Then the perovskite solution was spin coated onto the substrate at 5000 rpm for 30 s resulting in films with thicknesses of ≈50 nm and ≈130 nm for MAPbI_3_ and (BA)_2_(MA)Pb_2_I_7_, respectively. Next, a phenyl-C61-butyric acid methyl ester (PCBM) solution with a concentration of 20 mg/ml in toluene was spin coated directly on the perovskite film to form a thin electron transport layer of ≈20 nm-thick. Finally, a 50 nm-thick Au top electrode was deposited through a shadow mask using an electron beam evaporator at a rate of 0.5 A/s in a base pressure of ≈1 × 10^−6^ mbar. The LED active area is ≈1 mm^2^. We note that we used the Au electrode as an electron-injector in order to maximize the applied electric field on the devices^46^. But such a structure commonly yields a low EL quantum efficiency. The band diagrams of the devices are shown in the insets of Fig. 2b,c.

The devices were transferred to an optical helium closed-cycle cryostat with variable temperatures that was then placed in between the pole pieces of an electromagnet that produced magnetic fields *B* up to 3 kG with a one-Gauss resolution. The devices were driven at constant *V* using a Keithley 2400 source-meter unit and the EL intensity was simultaneously measured by a Si photo-detector, while sweeping *B*. The MC and MEL are defined as follows:

$$\begin{array}{c}MC=\frac{I(B)-I(0)}{I(0)}100 \% \\ MEL=\frac{EL(B)-EL(0)}{EL(0)}100 \% \end{array}$$

where I(B) and EL(B) are the device current and electroluminescence in the presence of a magnetic field *B*, respectively.

## Supplementary information

Supplementary Information.
