# Supplementary material for: Origin of Rashba Spin-Orbit Coupling in 2D and 3D Lead Iodide Perovskites
Source: Sci Rep. 2020 Mar 18;10:4964. doi: 10.1038/s41598-020-61768-8 (PMC7080819; doi:10.1038/s41598-020-61768-8)
Supplement: Supplementary file 1 — Supplementary Information. [file 41598_2020_61768_MOESM1_ESM.docx]

Supporting Information

Origin of Rashba Spin-Orbit Coupling in 2D and 3D Lead Iodide Perovskites

Minh T. Pham, Eric Amerling, Hoang M. Luong, Huy T. Pham, George K. Larsen, Luisa Whittaker-Brooks and Tho D. Nguyen*

Minh T. Pham, Hoang M. Luong, Tho D. Nguyen*

Department of Physics & Astronomy, University of Georgia, Athens, GA, 30602, USA

Eric Amerling and Luisa Whittaker-Brooks

Department of Chemistry, University of Utah, Salt Lake City, UT 84112

Huy T. Pham

Department of Materials Science and Engineering, Phenikaa University, Ha Dong, Hanoi 10000, Vietnam

George K. Larsen

National Security Directorate, Savannah River National Laboratory, Aiken, South Carolina, 29808, USA

Corresponding Author

Dr. Tho D. Nguyen*

Department of Physics & Astronomy, University of Georgia, Athens, GA, 30602, USA

[email:ngtho@uga.edu](mailto:email%3Angtho@uga.edu)

**1. XRD measurement and crystal grain size calculation**

We used a X-ray diffractometer with a CuK_α_ X-ray source (λ = 0.154nm). The Bragg scattering bands were translated into crystal plane-spacing, d_hkl_ using the Bragg relation *2d_hkl_sin(2θ)=λ*, where 2θ is the scattering angle and $d_{hkl}=\frac{a}{\sqrt{h^{2}+k^{2}+l^{2}}}$,where a is the cubic lattice parameter, and *hkl* are the cubic Miller indices. The nanocrystal grain size, D, was estimated using Scherrer’s equation:

$$D=\frac{0.9\lambda}{\Delta_{2\theta}\cos(\theta)}$$

Where $\Delta_{2\theta}$is the Full width at half maximum (FWHM) of the Bragg band at angle 2θ.


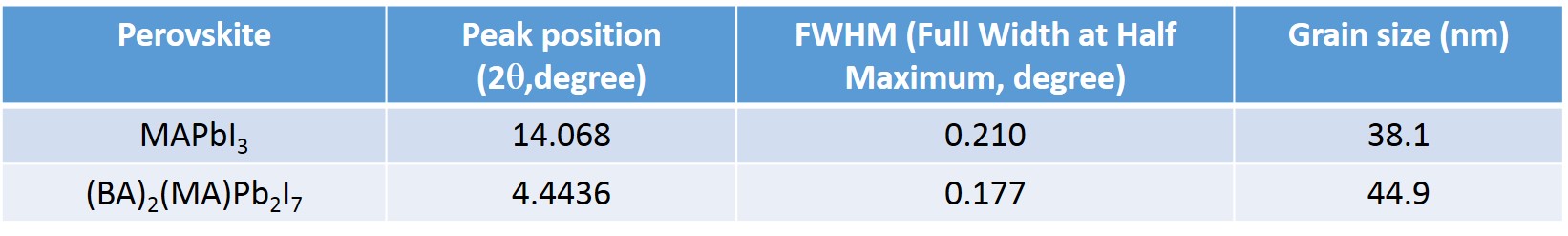


**Table S1.** Crystal grain size calculation for MAPbI_3_ and (BA)_2_(MA)Pb_2_I_7_  thin films


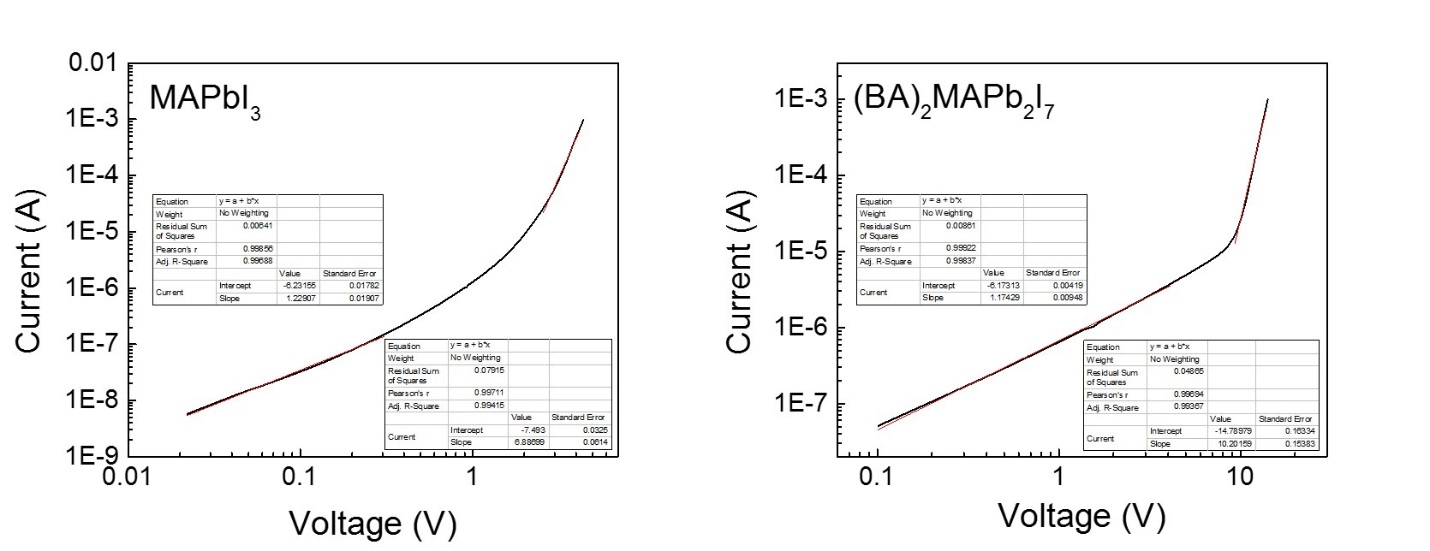


b)

a)

**Figure S1.** Current-voltage characteristics presented in (a) Figure 2b and (b) Figure 2c in the main text plotted in log-log scale. The Ohmic response (I ~ V^n^ with n ~ 1) is found when the applied voltages is larger than the EL turn-on voltages. At larger applied voltage, the electron-hole recombination yields a n = 6.8 for MAPbI3 and n = 10.2 for (BA)_2_(MA)Pb_2_I_7_ thin films.


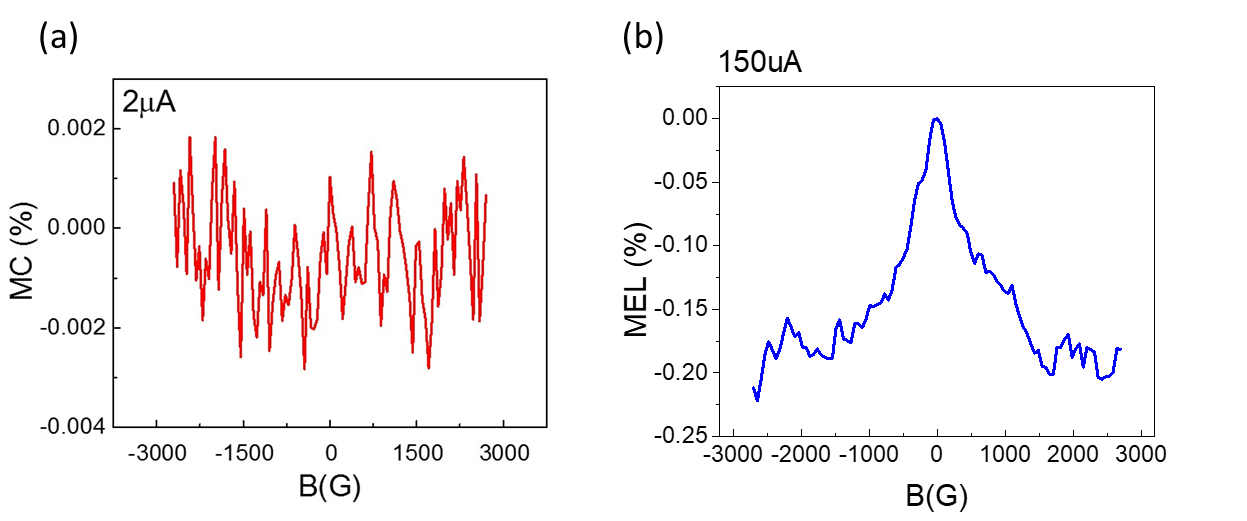


**Figure S2.** (a) Magnetoconductance (MC) of 3D MAPbI_3_ LED at a current density of 2 μA (without electroluminescence). (b) Magnetoelectroluminescence (MEL) of 3D MAPbI_3_ LED at a current density of 150 μA.


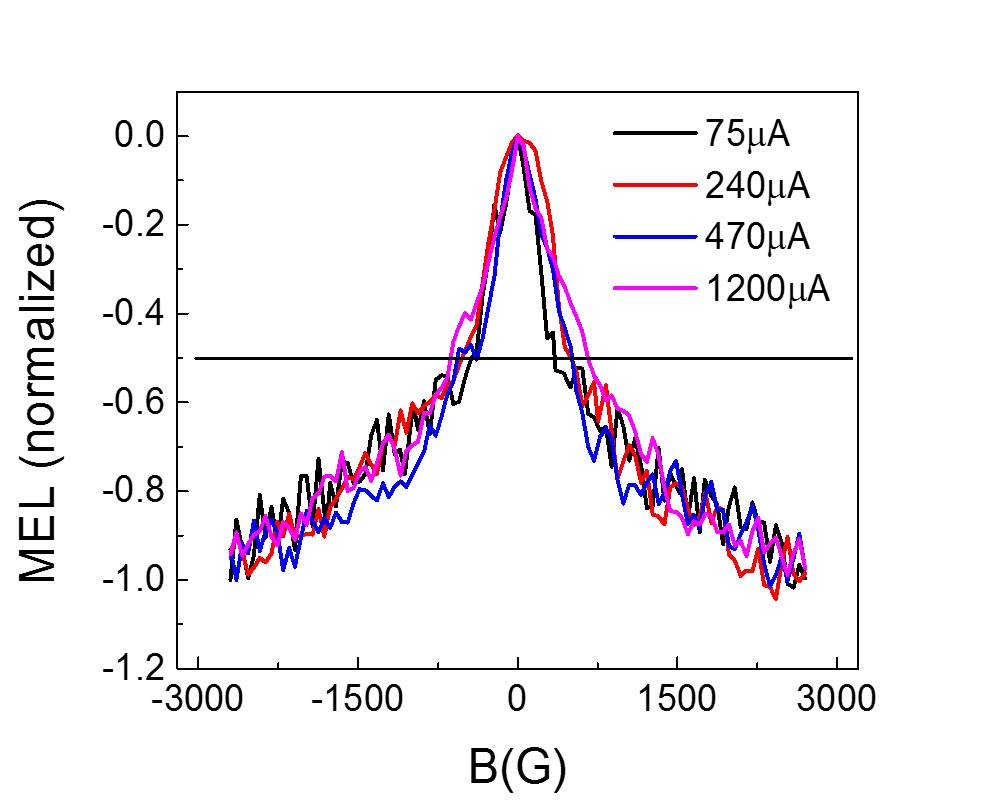


**Figure S3.** Normalized MEL responses at different current densities. The HWHM of the MEL in **Figure 3d**, main text, is read directly from the MEL responses.


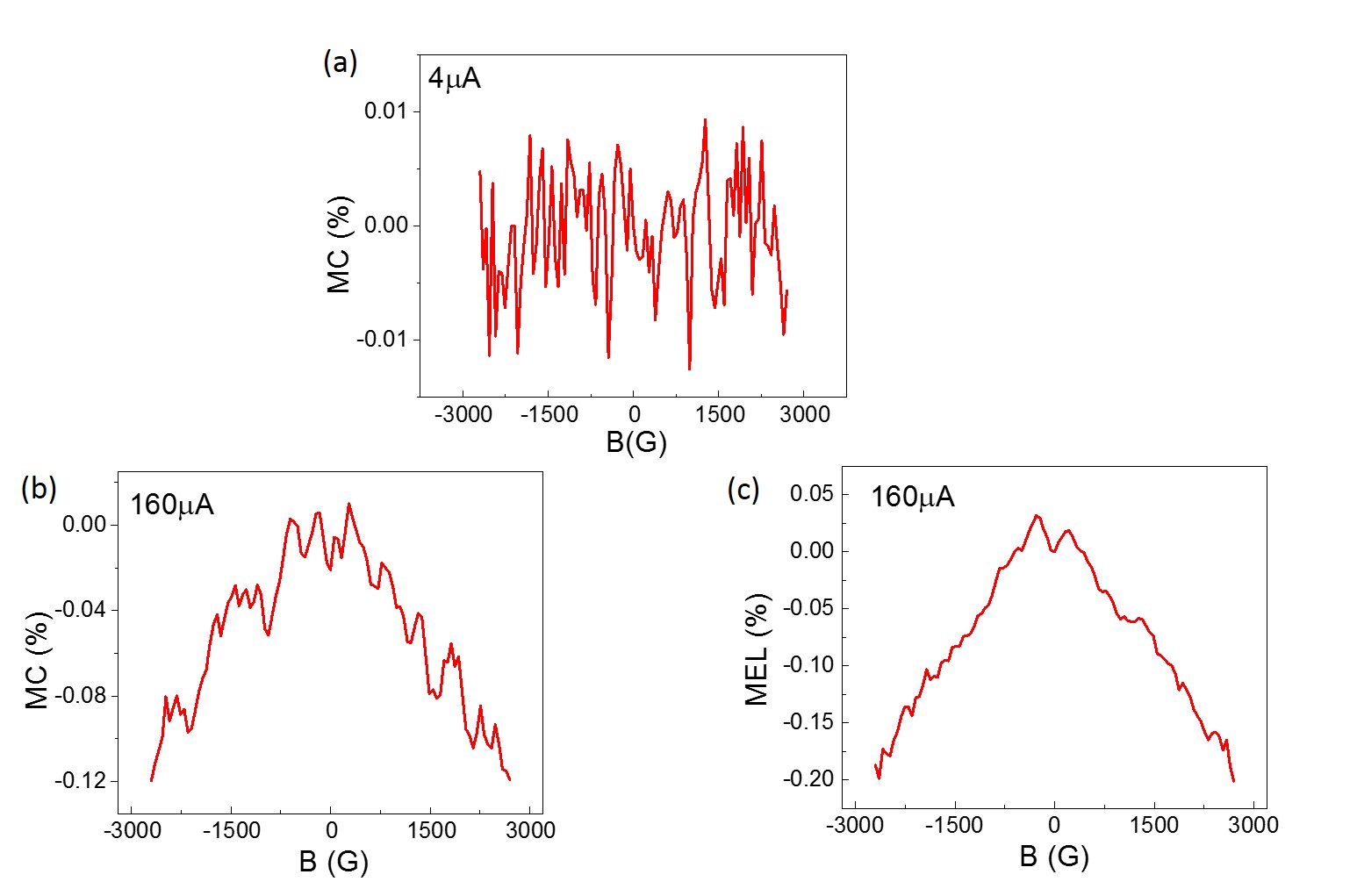


**Figure S4.** (a) Magnetoconductance (MC) of 2D (BA)_2_(MA)Pb_2_I_7_ LED at a current density of 4 μA (without Electroluminescence). (b) MC and (c) MEL of the device at a current density of 160 μA.


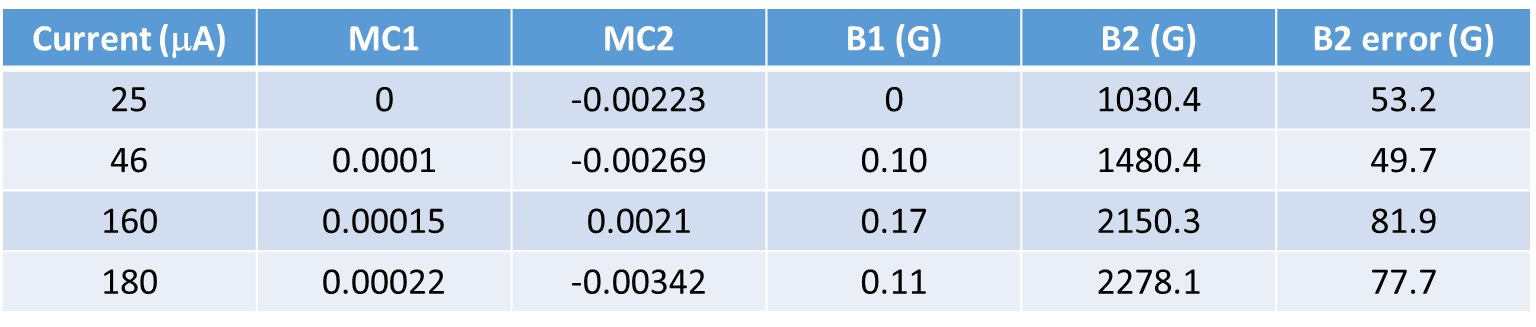


**Table S2**. Fitting parameters of the 2D Perovskite LED’s MEL responses.


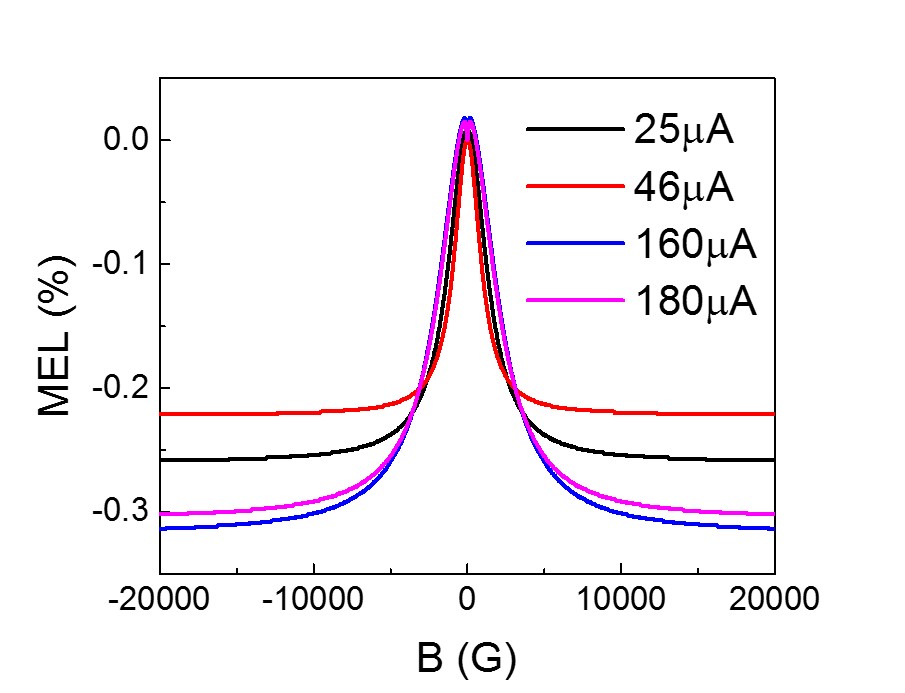


**Figure S5:** MEL responses obtained from the fits of the experimental MEL responses as such in Figure 3b. The data is plotted up to 20 kG. The HWHM of MEL in Figure 4d is read directly from those MEL curves.


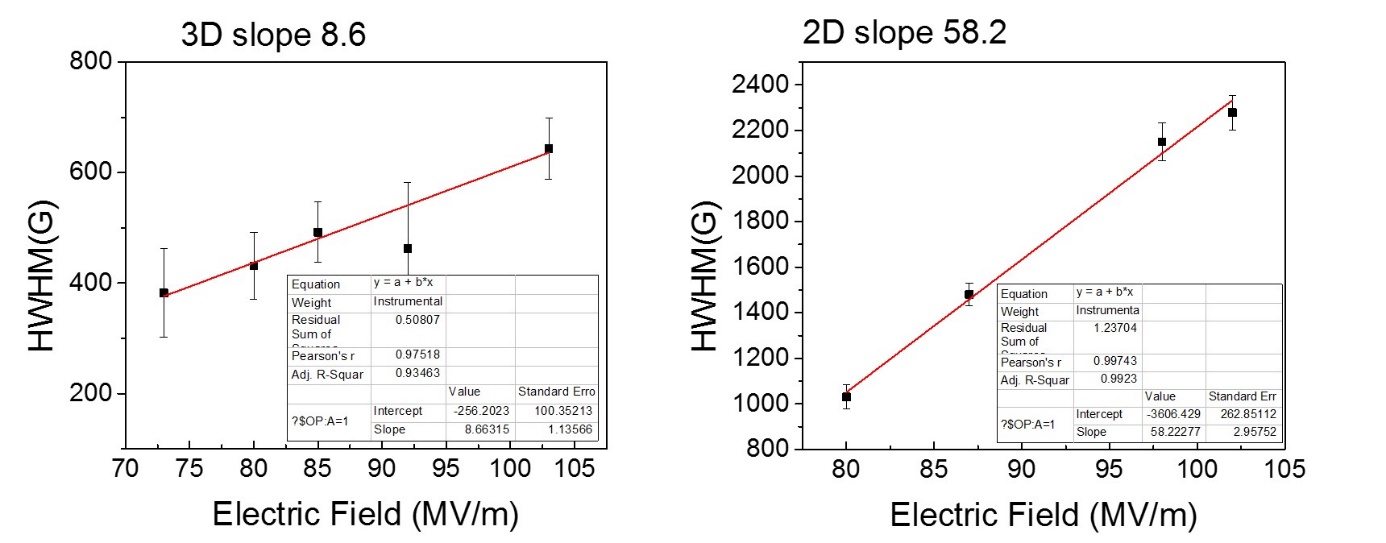


**Figure S6:** The HWHM of the MEL responses shown in Figures 3d and 4d as a function of the external electric field. The linear fits and the fitting parameters are shown for clarity.
